# H3K9 Demethylation-Induced R-Loop Accumulation Is Linked to Disorganized Nucleoli

**DOI:** 10.3389/fgene.2020.00043

**Published:** 2020-02-06

**Authors:** Hong Zhou, Le Li, Qing Wang, Yan Hu, Weiwei Zhao, Mayank Gautam, Lijia Li

**Affiliations:** State Key Laboratory of Hybrid Rice, College of Life Sciences, Wuhan University, Wuhan, China

**Keywords:** multiple nucleoli, H3K9 dimethylation, R-loops, ribosomal DNA, transcription inhibition

## Abstract

The nucleolar structure and integrity are important for a range of cellular functions of the nucleoli. It has been shown that cells lacking histone H3 Lysine 9 (H3K9) methylation form fragmented nucleoli. However, the molecular mechanism involved remains poorly understood. Here, we present evidence suggesting that loss of H3K9 dimethylation (H3K9me2) triggers R-loop accumulation at the rDNA locus, which further leads to the multilobed nucleoli. We reveal that suppression of H3K9 methyltransferase G9a by the inhibitor BIX 01294 causes R-loop accumulation at the rDNA region as well as inducing formation of multiple nucleoli. SiRNA-mediated knockdown of RNase H1 which can hydrolyze the RNA chain in R-loops causes an increase in R-loop formation, which in turn results in multiple nucleoli in one nucleus, whereas H3K9me2 levels are not affected by R-loop accumulation. Inhibition of RNA polymerase I transcription elongation by small molecule inhibitors induces a substantial decrease in H3K9me2 levels, accumulation of R-loops at rDNA sites, and nucleolus fragmentation. These results provide a mechanistic insight into the role of H3K9me2 in the structural integrity and organization of nucleoli via regulating R-loop accumulation.

## Background

The nucleolus is a prominent nuclear compartment formed around actively transcribed ribosomal DNA (rDNA). In the diploid human genome, approximately 400 copies of the rDNA repeats are tandemly arranged in specialized domains on the short arms of chromosomes 13, 14, 15, 21, and 22, which are termed as NORs. The rDNA is transcribed by RNA polymerase I (Pol I) into a 47S rRNA precursor transcript which is then processed into mature 28S, 5.8S, and 18S rRNAs. These mature rRNAs are a requisite for the assembly of the ribosomes in nucleoli. Only half of rDNA repeats are active within a cell and rRNA synthesis is regulated by altering the rate of transcription of individual repeats, or by changing the number of active genes (Russell and Zomerdijk, 2006; Grummt, 2010). The rRNA transcription abundance controls ribosome biogenesis and thus influences protein synthesis capacity, which regulates the cell growth and division rate in response to cellular stimuli (Mayer and Grummt, 2006). It has been shown that an increase in rRNA gene transcription is related to excessive protein synthesis which is significant for uncontrolled cancer cell proliferation and results in enlarged nucleoli (Andersen et al., 2005). Disruption of one or more of the steps that control ribosome biosynthesis could affect nucleolar structures (Schoefl, 1964). Recent studies have shown that the nucleolus performs several other functions during the cell cycle, development, and pathology (Sirri et al., 2008; Derenzini et al., 2009), and thus the normal structure of the nucleolus is apparently significant for its plurifunctionality.

Epigenetic mechanisms have been shown to regulate the transcription and structure of rDNA responding to environmental and developmental cues (Mcstay, 2006; Moss et al., 2007). The nucleolar remodeling complex NoRC was found to regulate rRNA expression by recruiting DNA methyltransferase and histone deacetylase to regulatory regions (Santoro et al., 2002). It has been shown that in DNA methyltransferase 1 (Dnmt1) deficient cells, the loss of DNA methylation and chromatin changes at the rDNA region is observed and is associated with a structurally disorganized nucleolus (Espada et al., 2007). Some studies have demonstrated that histone deacetylase Rpd3 mediates rDNA silencing and that deacetylase Sir2p regulates the nucleosome and chromatin structure at the rDNA region in yeast (Buck et al., 2002; Oakes et al., 2006). Histone methyltransferase and H3K9me2 are associated with transcriptional repression (Berger, 2007; Kubicek et al., 2007). Fragmented nucleoli are found in *Su(var)* mutant cells and the H3K9 methylation and RNAi pathways are required for the normal organization of nucleoli in *Drosophila* (Peng and Karpen, 2007). However, the mechanism underlying H3K9me2 as a regulator for the maintenance of nucleolar structure remains largely unclear.

It has been demonstrated that DNA supercoiling upstream at the RNA polymerase transcription site is always negative and can be easily separated, thereby causing formation of an RNA/DNA hybrid strand between the generated nascent RNA and the non-coding DNA strand, whereas the coding DNA strand exists as a single-strand. These nucleic acid structures composed of the RNA/DNA hybrid and the displaced single-stranded DNA are called R-loops (Drolet, 2006). R-loops may affect chromatin architecture and genome stability. The stable R-loop can form when the transcriptional elongation complexes are blocked (Helmrich et al., 2011). Disturbances in processing pre-mRNA also leads to the formation of R-loops, which impedes replication fork progression thereby causing genomic instability (Gómezgonzález et al., 2009; Tuduri et al., 2009; Aguilera and Garcã-A-Muse, 2012). It has been observed that R-loops modulate genome dynamics by linking to histone H3 S10 phosphorylation and chromatin condensation (Castellanopozo et al., 2013), and mutation of H3K9me-depositing histone methylation transferase in *Caenorhabditis elegans* shows a possible link with increased R-loops in genomic repeated elements (Zeller et al., 2016). The R-loops accumulate at the CpG island in promoters of human genes (Ginno et al., 2012). R-loops may induce the formation of repressive chromatin marks to promote Pol II pause (Skourti-stathaki et al., 2014).

In this study, we focused on understanding the factors responsible for disorganized nucleoli. Here we found that a loss of H3K9me2 caused an increase in R-loop formation at the rDNA region, and established a link between the formation of R-loops and nucleolar disruption. Our investigations and observations of a possible connection between H3K9me2, R-loops, and nucleolar organization provide a novel insight in understanding nucleolus structural integrity.

## Materials and Methods

### Cell Culture

All cell lines used in this study were purchased from the China Center for Type Culture Collection. HeLa and A549 cells were grown in Dulbecco’s modified Eagle’s medium (DMEM). These cells were grown at 37°C in a humidified atmosphere containing 5% CO_2_ supplemented with 10% fetal bovine serum, penicillin (20 units/ml), and streptomycin (20 units/ml).

### Drug Treatment

Stock solutions were prepared by dissolving ActD (Amersco, SF, United States) in dimethyl sulfoxide (DMSO), BMH21 (Selleck, Shanghai, China) in DMSO, CX5461 (Selleck, Shanghai, China) in dimethyl fumarate (DMF) and BIX 01294 (Selleck, Shanghai, China) in DMSO, respectively. Stock solutions were stored at −20°C and diluted to the respective experimental concentrations with phosphate buffer saline (PBS) prior to use.

### Flow Cytometry (FCM) Analysis

Quantification of drug treated cells was performed using the Annexin V-FITC detection kit (Beyotime, Shanghai, China) according to the instruction manual and the previously published protocol (Yan et al., 2009). After treatment with ActD, BMH21 and CX5461 for 24 h, the cells were collected and washed once with pre-chilled PBS. The cells were gently resuspended in the binding solution, followed by the addition of 5 μl Annexin VFITC and 10 μl propidium iodide (PI) dye liquor. The cell suspension was then mixed gently and incubated for 20 min at room temperature in the dark. The cells were re-suspended 2–3 times during the incubation to improve staining. After the completion of the dyeing, the sample was filtered through a 400-mesh screen to perform the FCM test. FITC Annexin-V staining was detected in the FL1 channel, whereas PI staining was monitored in the FL2 channel. Data were analyzed with Summit software.

### Transfection

The siRNA against Human RNase H1 and the negative control sequence were synthesized by GenePharma (Suzhou, China). The siRNA sequence for the Human RNase H1 is 5′-GGAUGGAGAUGGACAUGAA-3′ and the negative control sequence is 5′-UUCUCCGAACGUGUCACGUTT-3′ (Ruhanen et al., 2011; Zhou et al., 2012). The sequences for the RNase H1 protein were synthesized by Genewiz (Suzhou, China) and were cloned into pcDNA 3.0 plasmids. HeLa cells cultured in 6-well plates were transfected with 100 nM siRNAs for the RNase H1 RNA interference study, or 2 μg recombinant pcDNA3.0 plasmids for overexpressing RNase H1 proteins using the Lipofectamine 2000 reagent (Invitrogen, Carlsbad, CA, United States). Western blot analysis was performed to determine the RNase H1 protein contents with the anti-RNase H1 antibody. Cells were harvested 48 h after the transfection for western blot, immunostaining and ChIP.

### Western Blot Analysis

Whole-cell protein extracts were prepared from HeLa cells lysed in extraction buffer (100 mM Tris–HCl pH 7.4, 50 mM NaCl, 5 mM EDTA, and 1 mM PMSF). Proteins were loaded onto a 12% SDS-PAGE gel and separated by electrophoresis. Then, proteins were transferred to PVDF membranes (Millipore, Billerica, MA, United States) and the membranes were incubated with 5% non-fat milk-TBS for 2 h at room temperature. Afterward, the immunoblots were incubated overnight with the following primary antibodies: anti-H3 antibodies (Abcam, Cambridge, United Kingdom, ab1791), anti-H3K9me2 antibodies (Abcam, Cambridge, United Kingdom, ab1220), anti-GAPDH antibodies (Beyotime, Shanghai, China), and anti-RNase H1 monoclonal antibodies (Abcam, Cambridge, MA, United States, ab56560). The secondary antibodies were horseradish peroxidase (HRP) labeled goat anti-mouse IgG (Beyotime, Shanghai, China, A0126) and HRP labeled goat anti-rabbit IgG (Beyotime, Shanghai, China, A3327). Immunoreactivity was determined using the ECL method (Bio-Rad, Hercules, CA, United States) according to the manufacturer’s instructions.

### Immunofluorescence Staining

Immunofluorescence staining was performed as previously described (Huang et al., 2008; Gao et al., 2016). The cells were fixed on a glass slide with 4% paraformaldehyde for 5 min. Cells were washed three times with PBS for 5 min each. The cells were permeabilized with PBS containing 0.5% Triton-100 for 25 min and washed three times with PBS for 5 min each. Cells were blocked with 3% BSA for 60 min at 37°C and then washed three times with PBS for 5 min each. The primary antibody was diluted 1:100 with 0.5% BSA and incubated overnight at 4°C. Then, cells were washed three times with PBS for 5 min each. The Cy3- and the FITC-labeled secondary antibodies were diluted 1:100 with 0.5% BSA and the cells were incubated for 2 h at 37°C. The cells were washed three times with PBS for 5 min. 20 μl of 0.5 μg/ml DAPI was added to each slide for microscopic examination.

### Silver Staining

Silver staining of cells was carried out according to the previously described protocol (Howell and Black, 1980; Huang et al., 2012). Briefly, for the selective staining of Ag-NORs, two drops of the colloidal developer and four drops of aqueous silver nitrate were pipetted onto a glass slide containing cells and covered with a cover glass. The silver-staining mixture turned yellow after the slide was cultured at 70°C for 30 s, and within 2 min, it became golden-brown. Then, the staining mixture was rinsed off with deionized water. The slide was blotted dry and was examined immediately. The nucleolus organizer regions were stained black, while nuclei were stained yellow.

### Reverse Transcription and Real-Time PCR

Total RNA was isolated using a TRIzol reagent (Invitrogen, Carlsbad, CA, United States). The purified RNA was reverse-transcribed to cDNA using a Revert Aid First Strand cDNA Synthesis kit (Thermo Fisher Scientific, Waltham, MA, United States). qRT-PCR was performed using SYBR^®^ Green Real-time PCR Master Mix (Toyobo, Tokyo, Japan) in a StepOne Plus real-time PCR system (Applied Biosystems, Carlsbad, CA, United States). The amplification conditions were as follows: 94°C for 2 min, followed by 40 amplification cycles at 94°C for 5 s, 56°C for 15 s and 72°C for 20 s. Fluorescence data were acquired at the 72°C step and during the melting-curve program. The human glyceraldehyde-3-phosphate dehydrogenase (*GAPDH*) gene was used as a quantitative control for the amplified product, and the primer sequences were obtained according to the previously published data (Cong et al., 2012; Uemura et al., 2012).

### Chromatin Immunoprecipitation (ChIP)

Chromatin Immunoprecipitation was performed with anti-H3 antibodies (Abcam, Cambridge, United Kingdom, ab1791), anti-H3K9ac antibodies (Abcam, Cambridge, United Kingdom, ab12179), anti-H3K9me2 antibodies (Abcam, Cambridge, United Kingdom, ab1220), anti-H4K5ac antibodies (Abcam, Cambridge, United Kingdom, ab51997), anti-DNA-RNA Hybrid antibodies (Kerafast, Boston, MA, United States), following the procedure used by Cong et al. (2012). Rabbit serum was used as a negative control for mock immunoprecipitation. Precipitated genomic DNA was subjected to quantitative PCR with primers below that were designed to amplify approximately 200–1000 bp fragments encompassing the promoter region, the exon region and the intergenic spacer (IGS) region of the rDNA gene according to the real-time PCR procedure mentioned above. All primer sequences for ChIP were referenced from published data (Cong et al., 2012).

### Statistical Analysis

The data and error bars were calculated from three independent experiments. The data in this study were analyzed for significant differences between the experimental groups and control groups using the *t*-test. The results were considered statistically significant when *P* < 0.05.

## Results

### BIX Treatment Induces H3K9me2 Reduction and the Formation of Multiple Nucleoli in Mammalian HeLa Cells

**FIGURE 1:**
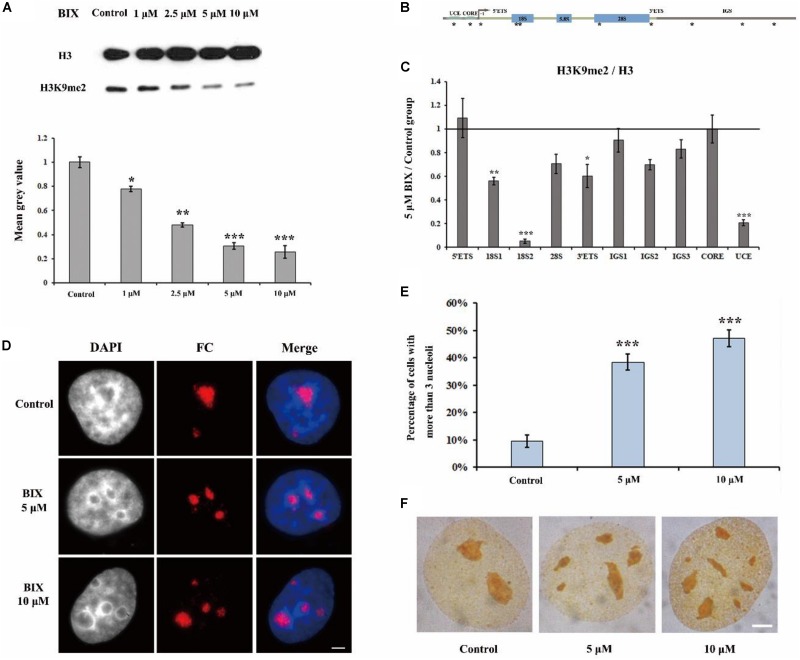
The effect of G9a inhibitor BIX on nucleoli of HeLa cells. **(A)** Western blot analysis of H3K9me2 in HeLa cells after treatment with BIX for 48 h. **(B)** Schematic representation of the human rRNA genes and the positions of analyzed amplicons. **(C)** ChIP analysis of H3K9me2 levels at the rDNA regions in HeLa cells after treatment with or without BIX for 48 h. The *y*-axis indicates the ratio of the relative quantities of rDNA in the experimental group HeLa cells to the relative quantities of rDNA in control group HeLa cells. The *x*-axis indicates different regions of rDNA amplicons. Relative values were normalized to those of the total H3. **(D)** HeLa cells were incubated with or without 5 μM or 10 μM BIX for 48 h and then indirect immunofluorescence staining with the anti-fibrillarin antibody was used to detect the nucleoli. Scale bar = 3 μm. **(E)** Percentages of interphase nuclei with over three fragmented nucleoli after treatment with or without 5 μM or 10 μM BIX for 48 h, respectively. The number of evaluated nuclei in each group was 300. **(F)** Ag-NOR staining was used to detect the nucleolar structure in HeLa cells after treatment with or without BIX for 48 h. Scale bar = 3 μm. Data are expressed as *P < 0.05, **P < 0.01, and ***P < 0.001, measured by the *t*-test.

It has been reported that in H3K9me2 defective drosophila cells, the nucleoli are disorganized (Peng and Karpen, 2007). In order to investigate the mechanism underlying H3K9me2 as a regulator for maintenance of nucleolar structure, we selectively removed the H3K9me2 modification by using the chemical inhibitor of H3K9 methyltransferase G9a, BIX-01294 (BIX); BIX can induce transient reduction of H3K9me2 in mammalian chromatin (Kubicek et al., 2007). Treatment of HeLa cells with BIX decreased the global H3K9me2 level in the genome (Figure 1A). Specifically, the level of H3K9me2 at rDNA regions was obviously decreased, as shown by ChIP analysis (Figures 1B,C). The normal interphase HeLa cell generally contains one to three nucleoli, detected by using immunofluorescence staining with an antibody against the nucleolus marker fibrillarin, which is a nucleolar protein participating in pre-rRNA processing. In the BIX-treated HeLa cells, the nucleolar structure was fragmented (Figure 1D) and the percentage of nuclei with more than three nucleoli was substantially increased (Figure 1E). Fragmented nucleoli were further verified by Ag-NOR staining signals in BIX treated cells (Figure 1F). These results indicated that H3K9me2 levels at rDNA sites are important for HeLa cells to maintain the normal nucleolar structure, as well as for insect drosophila cells (Peng and Karpen, 2007).

### R-Loops Are Involved in the Disruption of the Nucleolar Structure

**FIGURE 2:**
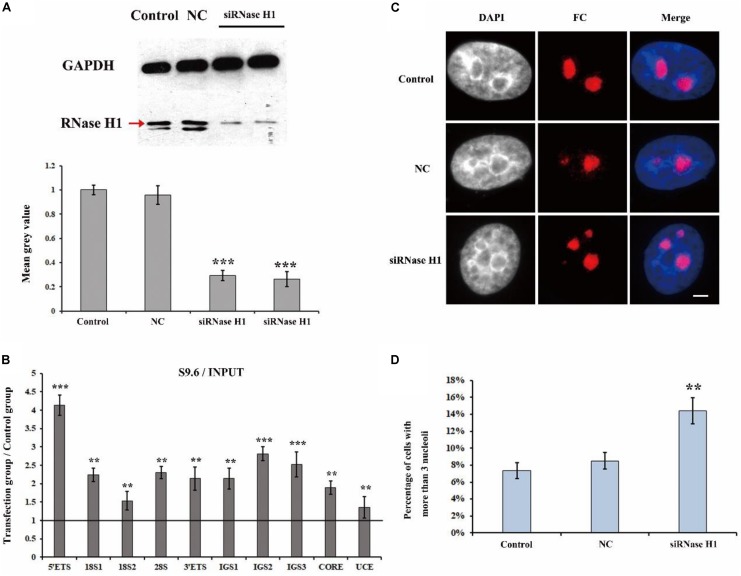
The effect of knockdown of RNase H1 on the R-loops and nucleoli of HeLa cells. **(A)** Western blot was used to detect the efficiency of the knockdown of RNase H1. **(B)** ChIP analysis of R-loops at the rDNA regions in HeLa cells after transfection with RNase H1 short-interfering RNA (siRNase H1) for 48 h. The *y*-axis indicates the ratio of the relative quantities of rDNA in the experimental group HeLa cells to the relative quantities of rDNA in control group HeLa cells. The *x*-axis indicates different regions of rDNA amplicons. Relative values were normalized to the input. Each experiment was repeated three times, and the average value and SD are shown. **(C)** HeLa cells were transfected with negative control siRNA (NC) or siRNase H1 for 48 h and then indirect immunofluorescence staining with the anti-fibrillarin antibody was used to detect the nucleoli. Scale bar = 3 μm. **(D)** Percentages of interphase nuclei with more than three fragmented nucleoli after transfection with siRNase H1 for 48 h. The number of evaluated nuclei in each group was 300. Data are expressed as *P < 0.05, **P < 0.01, and ***P < 0.001, measured by the *t*-test.

It has been reported that H3K9me2 impedes the formation of R-loops in repeat DNA sequences by suppressing transcription to stabilize and protect these DNA sequences in *C. elegans* (Zeller et al., 2016). The observation that a loss of H3K9me2 at the rDNA locus caused the disorganized nucleolar structure led us to consider that the level of R-loops may be involved in the formation of fragmented nucleolar structures. Therefore, we performed a series of experiments to investigate the possible relationship between R-loops and nucleolar structures. RNase H1 hydrolyzes the RNA chain in R-loops (Wahba et al., 2011) and a lack of RNase H1 impairs the decreasing of R-loops and results in further genome instability (Amon and Koshland, 2016). Thus, we first employed small interference RNA (siRNA) to knockdown RNase H1 in HeLa cells (Ruhanen et al., 2011). Our results showed a high efficiency of the knockdown of RNase H1 (Figure 2A). Moreover, ChIP analysis with anti-RNA-DNA hybrid antibodies (S9.6) showed that the reduction in RNase H1 led to significant R-loop accumulation at the rDNA region (Figure 2B). Strikingly, the immunostaining results with the fibrillarin antibody revealed that the decreased expression of RNase H1 triggered the formation of multiple nucleoli (Figure 2C), and the percentage of more than three fragmented nucleoli per nucleus was significantly increased (Figure 2D). These results indicated that the accumulations of R-loops led to disorganized nucleoli in HeLa cells.

### Reduction in H3K9me2 Induces Accumulation of R-Loops at the rDNA Region

**FIGURE 3:**
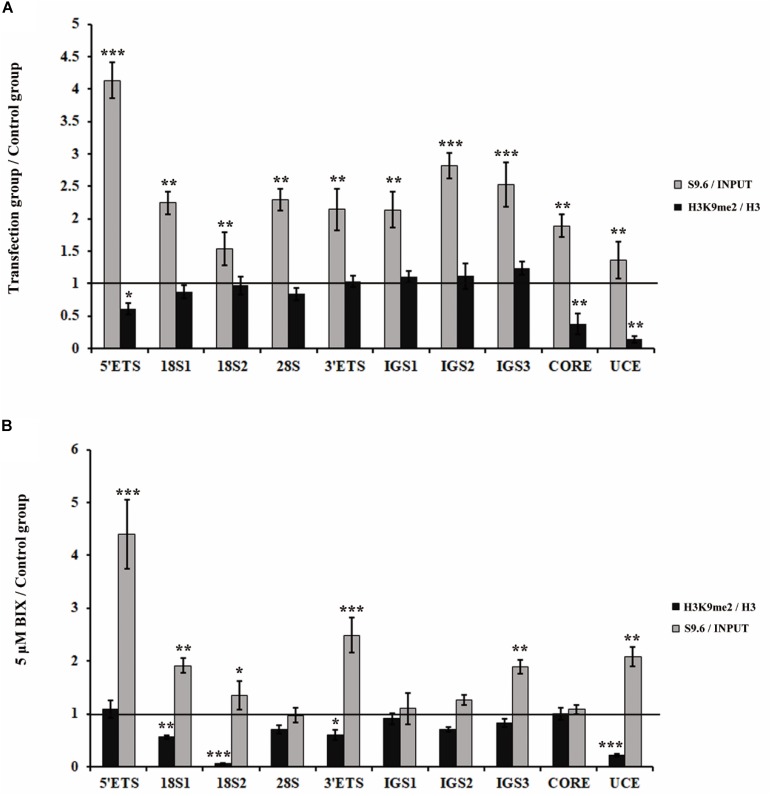
Analysis of the relationship between H3K9me2 and R-loops at rDNA sites in HeLa cells. **(A)** ChIP analysis showed the level of histone H3K9me2 and R-loops at the rDNA regions after transfection with siRNase H1 for 48 h. The *y*-axis indicates the ratio of the relative quantities of rDNA in the experimental group HeLa cells to the relative quantities of rDNA in control group HeLa cells. The *x*-axis indicates different regions of rDNA amplicons. Relative values of H3K9me2 were normalized to those of the total H3 and relative values of R-loops were normalized to the input. Each experiment was repeated three times, and the average value and SD are shown. **(B)** ChIP analysis of R-loops and the levels of H3K9me2 at the rDNA regions in HeLa cells after treatment with or without BIX for 48 h. Each experiment was repeated three times, and the average value and SD are shown. Data are expressed as *P < 0.05, **P < 0.01, and ***P < 0.001, measured by the *t*-test.

Considering that both the loss of H3K9me2 and the accumulations of R-loops induced fragmented nucleoli, we further determined whether H3K9me2 reduction is the cause or consequence of R-loop accumulation. First, we examined whether R-loops affected H3K9me2 levels at the rDNA region. As a result, H3K9me2 signals were unaffected by R-loop accumulation which was induced by the knockdown of RNase H1 at the rDNA region (Figure 3A), indicating that R-loops did not cause the variation in the H3K9me2 level. However, the reduction of the H3K9me2 level caused by BIX treatment triggered substantial accumulation in R-loop signals at the rDNA region (Figure 3B). These findings led us to hypothesize that the chromatin repressive mark H3K9me2 at the rDNA region was, at least part of, the regulatory factor of R-loops and that both of them regulate the nucleolus organization.

### H3K9me2 Is Also Involved in Transcription Inhibitor-Induced Nucleolar Structure Changes

**FIGURE 4:**
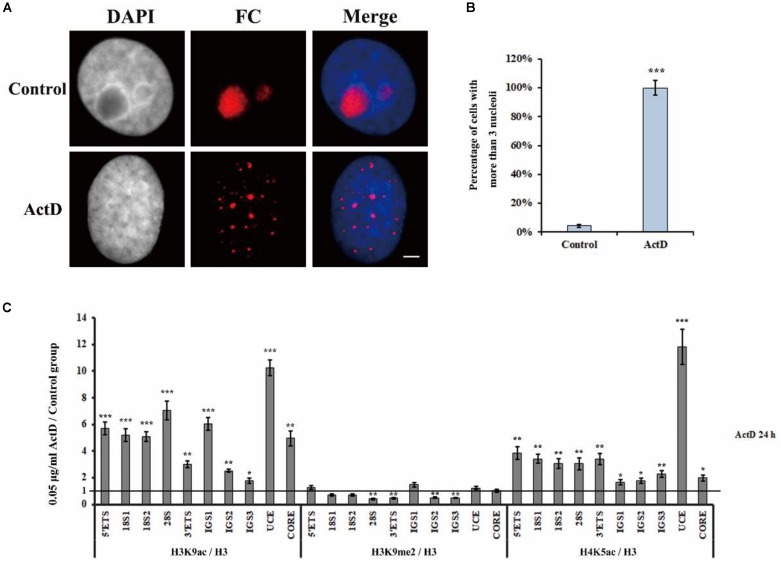
The changes of epigenetic modifications in rDNA site of HeLa cells after treatment with ActD. **(A)** HeLa cells were treated with or without ActD for 24 h and then indirect immunofluorescence staining with the anti-fibrillarin antibody was used to detect the nucleoli. Scale bar = 3 μm. **(B)** Percentages of interphase nuclei with over three fragmented nucleoli after treatment with or without ActD. The number of evaluated nuclei in each group was 300. **(C)** ChIP analysis of levels of H3K9ac, H3K9me2, and H4K5ac at the rDNA regions in HeLa cells after treatment with or without ActD. The *y*-axis indicates the ratio of the relative quantities of rDNA in the experimental group HeLa cells to the relative quantities of rDNA in control group HeLa cells. The *x*-axis indicates different regions of rDNA amplicons. Relative values were normalized to those of the total H3. Each experiment was repeated three times and the average values are shown with the SD. Data are expressed as *P < 0.05, ***P < 0.05, **P < 0.01, and ***P < 0.001, measured by the *t*-test.

Actinomycin D is an anti-tumor antibiotic and can inhibit pre-rRNA chain elongation through binding to the CpG-rich region at the actively transcriptional rDNA site (Harris, 1976). BMH21 also inhibits Pol I transcription by the formation of a stable complex with the CpG-rich DNA site (Peltonen et al., 2010). CX5461 specifically inhibits Pol I transcription of rRNA by selectively targeting the SL1 transcription factor (Drygin et al., 2011). Studies have shown that transcription inhibitors, such as ActD, BMH21 and CX5461, are able to affect nucleolar structure organization (Drygin et al., 2011; Mangan et al., 2017; Wei et al., 2018). Therefore, we conducted a series of experiments to investigate whether H3K9me2 and R-loops are involved in the transcription inhibitor-mediated nucleolar structure changes. Previously published data and flow cytometry were used to select the concentration of inhibitors according to their effects on HeLa cell apoptotic rates (Supplementary Figure S1A; Peltonen et al., 2014; Hong et al., 2017; Gelgor et al., 2018). The concentrations of transcription inhibitors used in this article were indicated in Supplementary Figure S1B. Apparently, transcription inhibition led to the formation of multiple nucleoli in HeLa cells and the percentage of nuclei with more than three nucleoli was substantially increased (Figures 4A,B and Supplementary Figures S2A,B). Similarly, these three inhibitors were also found to induce multiple nucleoli in human A549 cells (Supplementary Figure S3).

Levels of H3K9me2, Histone H3 Lysine 9 acetylation (H3K9ac) and H4 Lysine 5 acetylation (H4K5ac) at the rDNA locus of HeLa cells were analyzed using ChIP after treatment with indicated transcription inhibitors for 24 h (Figure 4C and Supplementary Figures S2C,D). It was interesting to observe that H3K9ac and H4K5ac, which are associated with active or open chromatin, were significantly increased, whereas the heterochromatin marker H3K9me2 was substantially decreased in all inhibitors treated HeLa cells. These results showed that transcription inhibitor-induced nucleolar structure changes were accompanied with decreasing levels of H3K9me2.

### Changes in the H3K9me2 Level Occurs Prior to Nucleolar Fission in HeLa Cells After ActD Treatment

**FIGURE 5:**
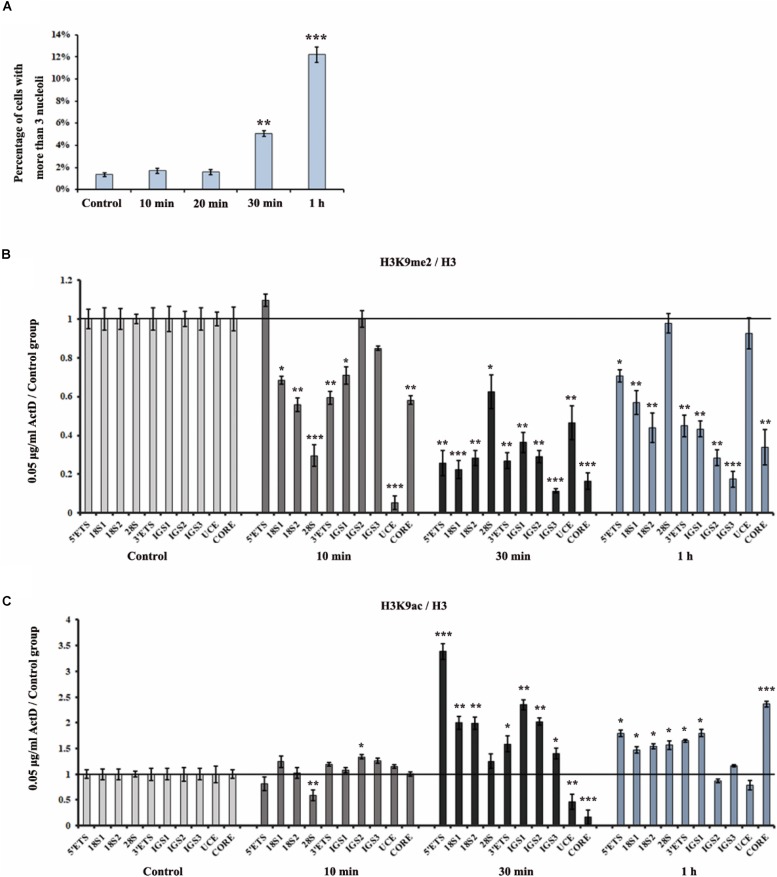
Analysis of disorganized nucleoli and histone modifications at rDNA regions in HeLa cells after treatment with ActD at different time points. **(A)** Percentages of interphase nuclei with over three fragmented nucleoli after treatment without or with ActD at different time points. The number of evaluated nuclei in each group was 300. **(B)** ChIP analysis of the level of H3K9me2 at the rDNA regions in HeLa cells after treatment with or without ActD at different time points. The *y*-axis indicates the ratio of the relative quantities of rDNA in the experimental group HeLa cells to the relative quantities of rDNA in control group HeLa cells. The *x*-axis indicates different regions of rDNA amplicons. **(C)** ChIP analysis of the level of H3K9ac at the rDNA regions in HeLa cells after treatment without or with ActD at different time points. Relative values were normalized to those of the total H3. Each experiment was repeated three times and the average values are shown with the SD. Data are expressed as *P < 0.05, **P < 0.01, and ***P < 0.001, measured by the *t*-test.

To analyze the effect of ActD on rDNA and nucleoli at different time points, we first measured the distribution of fibrillarin in the nucleus after treatment of HeLa cells with ActD for 10 min, 20 min, 0.5 h, and 1 h. The immunostaining examination showed that percentages of interphase nuclei with more than three fragmented nucleoli increased significantly after treatment for 0.5 h with ActD compared with the control, while no significant change was observed in ActD-treated cells for the time points lower than 0.5 h (Figure 5A). ChIP analysis of the levels of H3K9me2 and H3K9ac at the rDNA region in ActD-treated HeLa cells and the control was performed for different time points. H3K9me2 exhibited a significant decrease, whereas H3K9ac slightly increased, in HeLa cells treated with ActD for 10 min (Figures 5B,C). Our findings demonstrated that the changes of histone modification H3K9me2 occurred before nucleolar disorganization in chemical inhibitor-treated HeLa cells.

### Treatment With Transcription Inhibitors Also Triggers R-Loop Accumulation at rDNA Sites in HeLa Cells

**FIGURE 6:**
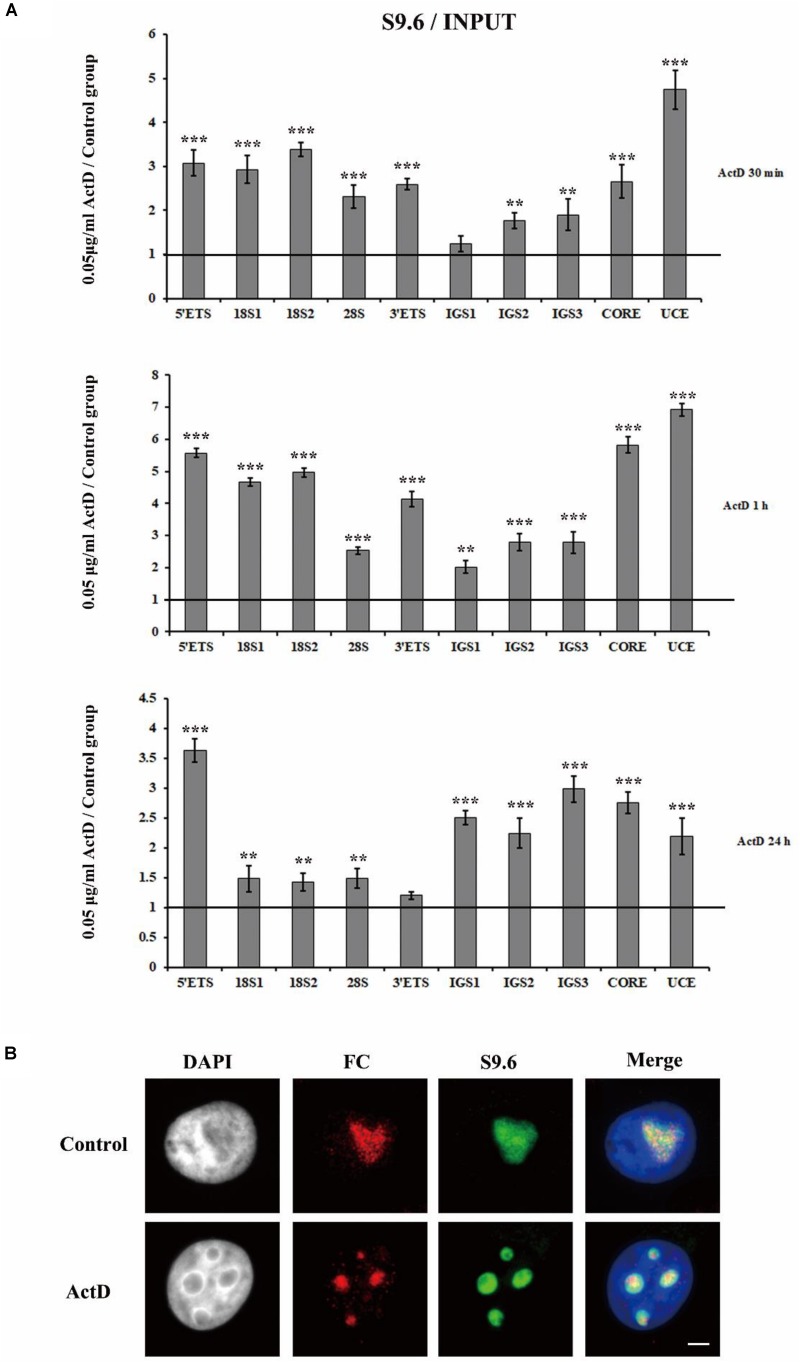
The accumulation and localization of R-loops in HeLa cells after treatment with Pol I transcription inhibitors. **(A)** ChIP analysis of R-loops at the rDNA regions in HeLa cells after treatment with ActD for 0.5 h, 1 h, and 24 h. The *y*-axis indicates the ratio of the relative quantities of rDNA in the experimental group HeLa cells to the relative quantities of rDNA in control group HeLa cells. The *x*-axis indicates different regions of rDNA amplicons. Relative values were normalized to the input. Each experiment was repeated three times and the average values are shown with the SD. **(B)** Nucleoli and R-loops were detected by indirect immunofluorescence staining with an antibody against fibrillarin (FC, red) and an antibody against R-loop (S9.6, green) in interphase nuclei of ActD-treated Hela cells at 1 h. Data are expressed as *P < 0.05, **P < 0.01, and ***P < 0.001, measured by the *t*-test.

To check if R-loops were also involved in the transcription inhibitor-mediated nucleolar structure changes, we next examined the level of R-loops at the rDNA locus in HeLa cells which were treated with transcription inhibitors for indicated times. The results of ChIP experiments with antibody S9.6 showed that R-loop signals were significantly enhanced in HeLa cells treated with ActD for 0.5 h, 1 h, and 24 h (Figure 6A). Furthermore, we confirmed the association of R-loops and nucleoli in single cells using immunofluorescence staining with the antibody S9.6 and the antibody against fibrillarin after treatment with inhibitors for 1 h. Our results indicated that the distribution of DNA-RNA hybrids and fibrillarin were co-localized (Figure 6B and Supplementary Figure S4), suggesting that R-loops were associated with fragmented nucleoli at the cellular level.

**FIGURE 7:**
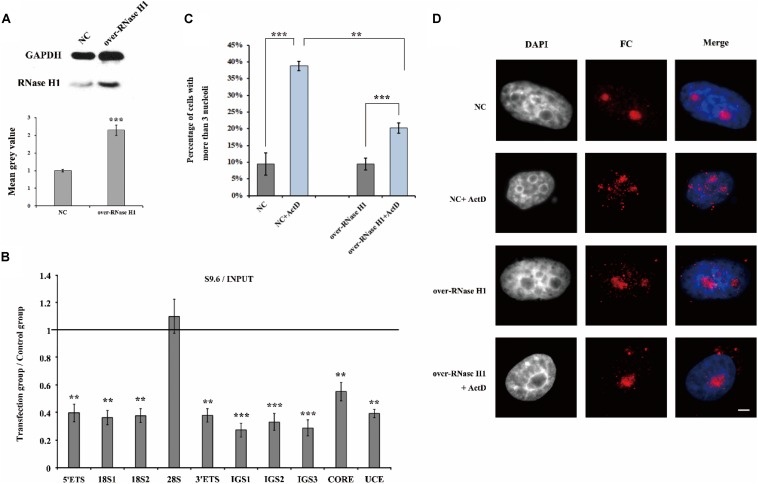
The effect of overexpression of RNase H1 on ActD-induced nucleolar fragmentation in HeLa cells. **(A)** Western blot was used to detect the efficiency of the RNase H1 overexpression plasmid. NC represents samples transfected with empty plasmids. Over-RNase H1 represents samples transfected with pcDNA3.0-RNase H1. **(B)** ChIP analysis of R-loops at the rDNA regions in HeLa cells after transfection with RNase H1 overexpression plasmid. The *y*-axis indicates the ratio of the relative quantities of rDNA in the experimental group HeLa cells to the relative quantities of rDNA in control group HeLa cells. The *x*-axis indicates different regions of rDNA amplicons. Relative values were normalized to the input. Each experiment was repeated three times and the average values are shown with the SD. **(C)** Percentages of interphase nuclei with more than three fragmented nucleoli after treatment with or without ActD for 24 h. Number of evaluated nuclei in each group was 300. **(D)** Nucleoli were detected by indirect immunofluorescence staining with an antibody against fibrillarin. Scale bar = 3 μm. Each experiment was repeated three times and the average values are shown with the SD. Data are expressed as *P < 0.05, **P < 0.01, and ***P < 0.001, measured by the *t*-test.

Furthermore, we overexpressed RNase H1 via transient transfection with a recombinant plasmid pcDNA3.0-RNase H1 in HeLa cells (Figure 7A) and as expected, the level of R-loops at the rDNA locus was decreased (Figure 7B). After ActD treatment, HeLa cells transfected with overexpressed RNase H1 showed lower levels of nucleolus fission than cells transfected with an empty vector (Figures 7C,D), indicating that a reduction of R-loop accumulation can effectively mitigate the effect of ActD on nucleolar structures.

### An Increase in rDNA Transcription Initiation Is the Possible Cause of R-Loop Accumulation at the rDNA Site

**FIGURE 8:**
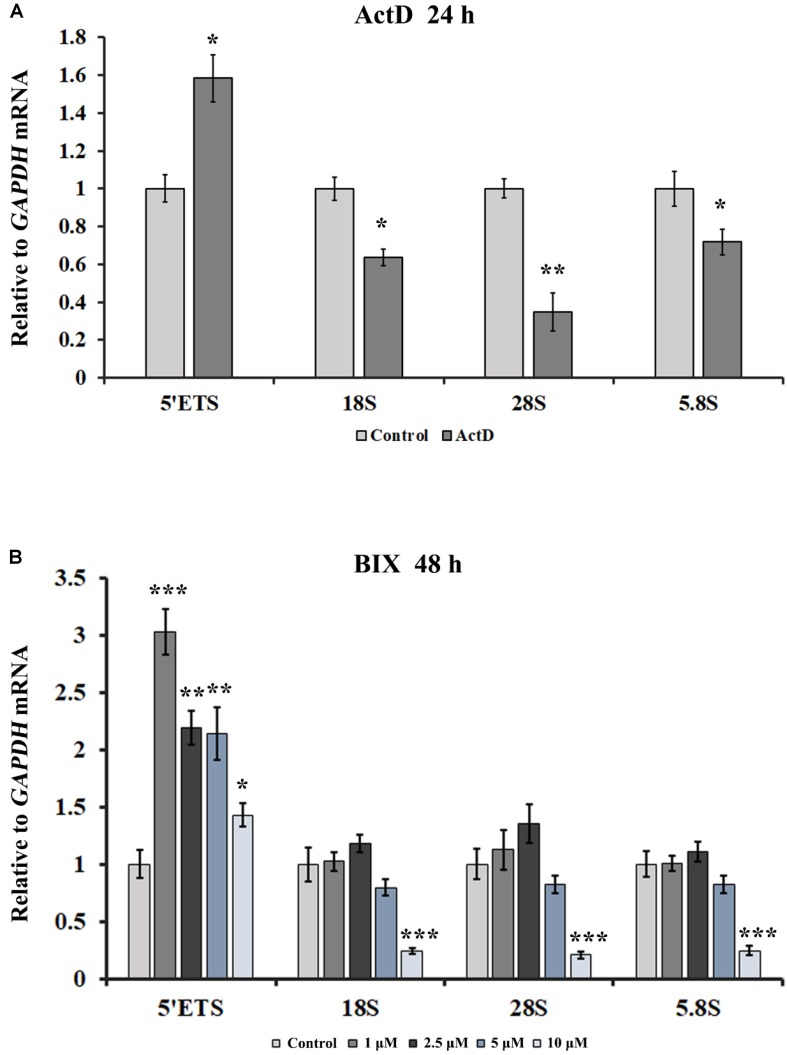
The effects of ActD and BIX treatment on rDNA transcription initiation. **(A)** qRT-PCR was used to detect the incomplete 5′ETS transcripts and the mature rRNA expressions in HeLa cells after treatment with ActD for 24 h. **(B)** qRT-PCR was used to detect the incomplete 5′ETS transcripts and the mature rRNA expressions in HeLa cells after treatment with BIX for 48 h. Expression values were normalized to the gene *GAPDH*. The relative expression ratio of each sample was compared with untreated cells, the expression value of which were assigned as 1. Each experiment was repeated three times, and the average value and SD are shown. Data are expressed as *P < 0.05, **P < 0.01, and ***P < 0.001, measured by the *t*-test.

Transcription is the primary pathway that gives rise to R-loops, and H3K9me2 is the marker of suppressive transcription. It is puzzling that transcription inhibitors led to an increase in R-loops. Here we used quantitative real-time PCR (qRT-PCR) to detect the transcription of rDNA genes in the presence of ActD and other transcription inhibitors. As shown in Figure 8A, the transcriptional levels of mature rRNA 18S, 28S, and 5.8S were partially inhibited under the presence of inhibitors for 24 h. However, the 5′ external transcribed spacer (5′ETS) expression level increased significantly after treatment with these inhibitors (Figure 8A and Supplementary Figure S5A), suggesting that rRNA gene transcription initiation was enhanced. It has been reported that ActD can stimulate the transcription initiation of rRNA genes (Hadjiolova et al., 1995). However, recent studies have shown that after treatment with BMH21 or CX5461 for a short time, such as 60 min or 15 min, the expression levels of short-lived 5′ETS were down-regulated (Drygin et al., 2011; Wei et al., 2018). This difference is likely to be due to the different cell lines, treatment times and concentrations used for these inhibitors. Additionally, up-regulation of rRNA gene transcription initiation was also observed in inhibitor-treated human A549 cells (Supplementary Figure S5B). Interestingly, treatment of HeLa cells with the epigenetic inhibitor BIX led to an increase in rDNA transcription initiation; however, the higher the concentration of BIX treatment, the lower the mature rRNA expression level was (Figure 7B).

## Discussion

**FIGURE 9:**
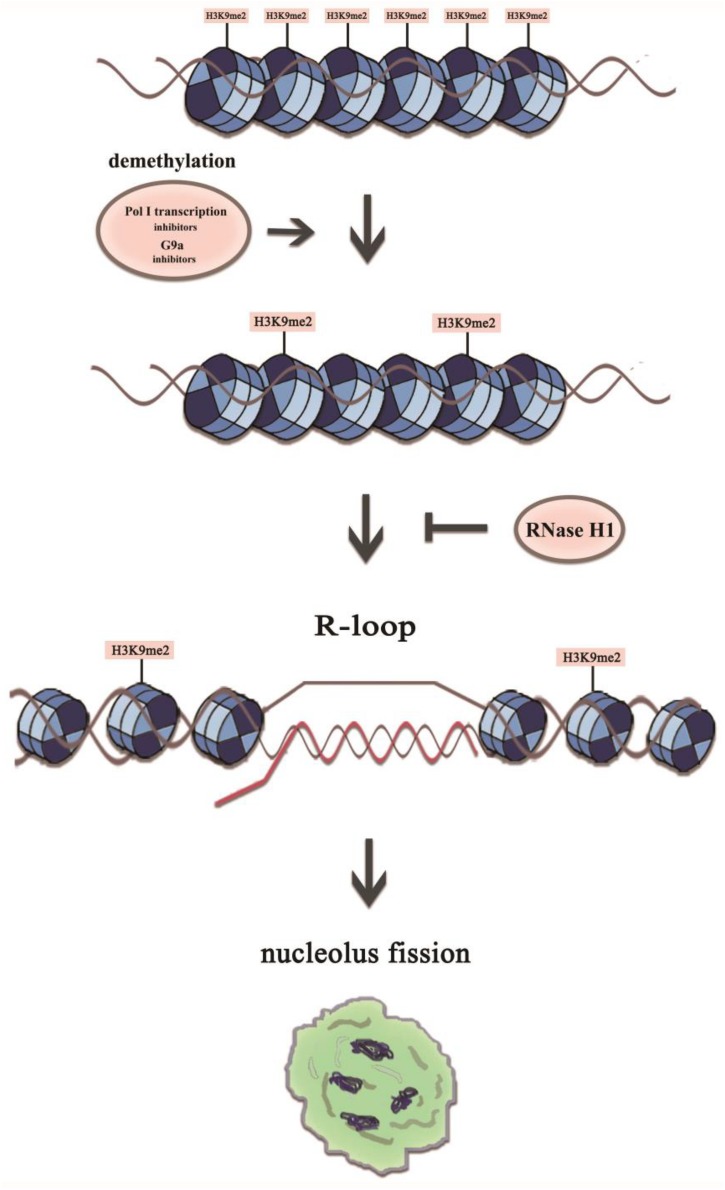
A model representing mechanistic link between H3K9me2 and nucleolus fission via the accumulation of R-loops at the rDNA regions. The transient R-loop formed in the transcriptional process is hydrolyzed by RNase H1, leading to complementation of template strand DNA and antisense strand. However, in the presence of RNA polymerase I transcription inhibitors or the epigenetic inhibitor BIX, the level of H3K9me2 decreases significantly, which leads to the augmentation of rDNA transcription initiation and R-loop accumulation, resulting in a structurally disorganized nucleolus.

The nucleoli are enriched with a large number of proteins with multiple functions besides ribosome biogenesis, and therefore the normal architecture of nucleoli is essential for a range of cellular functions (Frescas et al., 2007; Peng and Karpen, 2007). The nucleolar structure modifications may indicate that the adoption of new structures of nucleoli is to counter stress, to acquire sufficient rRNA and to maintain the normal cellular ribosomal level and physiological activity. What molecular intermediates may underlie the disturbance of nucleolus integrity? Here, we showed that a reduction in the H3K9me2 level led to an increase in the number of nucleoli *via* induction of R-loop accumulation in the nucleolus, and transcription inhibitor-mediated nucleolar structure changes were associated with H3K9me2 and R-loops at the rDNA locus in HeLa cells. The possible link between these factors is shown in Figure 9. Overall, our results provided new mechanistic insights into how H3K9me2 regulates nucleolar organization.

The nucleolar integrity is affected by epigenetic modifications. DNA methylation has been demonstrated to play an important role in the proper organization of nucleolar structures. For example, HCT116 cells lacking DNMT1 exhibited a disorderly nucleolar structure with small fragmented nucleoli (Espada et al., 2007). Gagnon-Kugler et al. showed that, in HCT116 cells, dismissing DNA methylation of RNA polymerase I promoter by double knockout of DNMT1 and DNMT3b resulted in altered rRNA synthesis and highly dispersed nucleoli which were represented as thread-like across the nucleus (Gagnonkugler et al., 2009). H3K9 methyltransferase and several other regulators of gene silencing are also required for the formation of normal nucleolus in drosophila (Peng and Karpen, 2007). In the present study, ChIP data showed that the methyltransferase inhibitor BIX was capable of causing partial mammalian HeLa cell nucleolar disruption, and a significant reduction of H3K9me2 at the rDNA region appeared prior to nucleolus fission in transcriptional inhibitor-treated HeLa cells. Therefore, we concluded that the epigenetic modification H3K9me2 at the rDNA locus was involved in the nucleolar structure maintenance in both drosophila and mammalian cells, and a decrease of H3K9me2 levels was sufficient to disrupt the integrated nucleolar structure.

It has been demonstrated that R-loops promote DNA methylation or histone modifications. RNA-DNA hybrids induce repressive chromatin marks over mammalian gene terminators (Skourti-stathaki et al., 2014), and R-loops are tightly linked to histone H3S10 phosphorylation in yeast cells (Castellanopozo et al., 2013), whereas formation of R-loops at CpG island promoters inhibits methylation and transcriptional silencing (Ginno et al., 2012). It has recently been reported that a loss of H3K9me2 and H3K9me3 in *C. elegans* is linked to R-loop enrichment at some genomic repeat elements; however, no significant increase in R-loop formation is observed at the rDNA locus in H3K9me-deficient mutant elegans (Zeller et al., 2016). Our results showed that BIX-treated HeLa cells exhibited a reduced level of H3K9me2 at the rDNA sites as well as enhanced R-loop accumulation, and treatment of HeLa cells with rDNA transcription inhibitors led to nucleolar fission accompanied with decreased H3K9me2 and increased R-loop accumulation at the rDNA region. However, H3K9me2 signals at the rDNA region were unaffected by the accumulation of R-loops due to knockdown of RNase H1 by siRNA. Therefore, these findings predicted that a decrease in the repressive mark H3K9me2 at the rDNA region triggered the accumulation of R-loops and subsequently led to nucleolar structure changes. Loss of H3K9me2 resulted in an increase of R-loops, presumably by enhancing transcription initiation at rDNA sites. This was supported by an increase in the rDNA transcription initiation induced by BIX, and strikingly, treatment of HeLa cells with transcription inhibitors also caused an increase in rDNA transcription initiation as well as partial inhibition of rDNA transcription elongation.

Some human cancers have changes in DNA methylation and histone modification at rDNA sites. For example, human hepatocellular carcinomas showed significant hypomethylation of the rDNA promoter, accompanied with an elevated rRNA synthesis rate (Huang et al., 2002). A low level of rDNA methylation is associated with up-regulation of rDNA expression in ovarian cancers and cervical cancers (Chan et al., 2005; Zhou et al., 2016). The increasing evidence suggests that overexpression of rRNA can be an initiating step in tumorigenesis (Drygin et al., 2010). Some approved cancer therapeutic strategies were reported to act through inhibition of rRNA synthesis, and selective inhibition of rDNA transcription has been demonstrated to be an effective strategy for the treatment of cancer (Drygin et al., 2010). Therefore, we highlighted the impact of histone modification and R-loop regulation in the nucleolus structure, and the use of targeting R-loops and rDNA transcription as an emerging cancer therapeutic strategy.

To obtain a more complete understanding of nucleolar disintegration, we must move beyond H3K9me2 to explore other factors in order to determine the link between nucleolar disruption and R-loops. Our present investigation will be instrumental for further exploration of the underlying mechanism of nucleolar disintegration. It shall be interesting to further elucidate how the nucleolus tolerates physiological R-loops and avoids their harmful effects, and to explore how the HeLa cells process them.

## Data Availability Statement

All datasets generated for this study are included in the article/Supplementary Material.

## Author Contributions

HZ, LeL, QW, YH, and WZ performed the experiments. LiL, HZ, LeL, QW, and YH planned and analyzed the experiments. HZ, LeL, QW, and YH helped with data analysis. LiL, HZ, LeL, QW, and MG wrote and edited the manuscript. LiL conceived the study.

## Conflict of Interest

The authors declare that the research was conducted in the absence of any commercial or financial relationships that could be construed as a potential conflict of interest.

## Abbreviations

ActD: actinomycin D
NORs: nucleolar organizing regions
siRNA: small-interference RNA
