# Supplementary material for: H3K9 Demethylation-Induced R-Loop Accumulation Is Linked to Disorganized Nucleoli
Source: Front Genet. 2020 Feb 6;11:43. doi: 10.3389/fgene.2020.00043 (PMC7025566; doi:10.3389/fgene.2020.00043)
Supplement: Supplementary file 1 [file Data_Sheet_1.pdf]

Supplementary Figures and legends

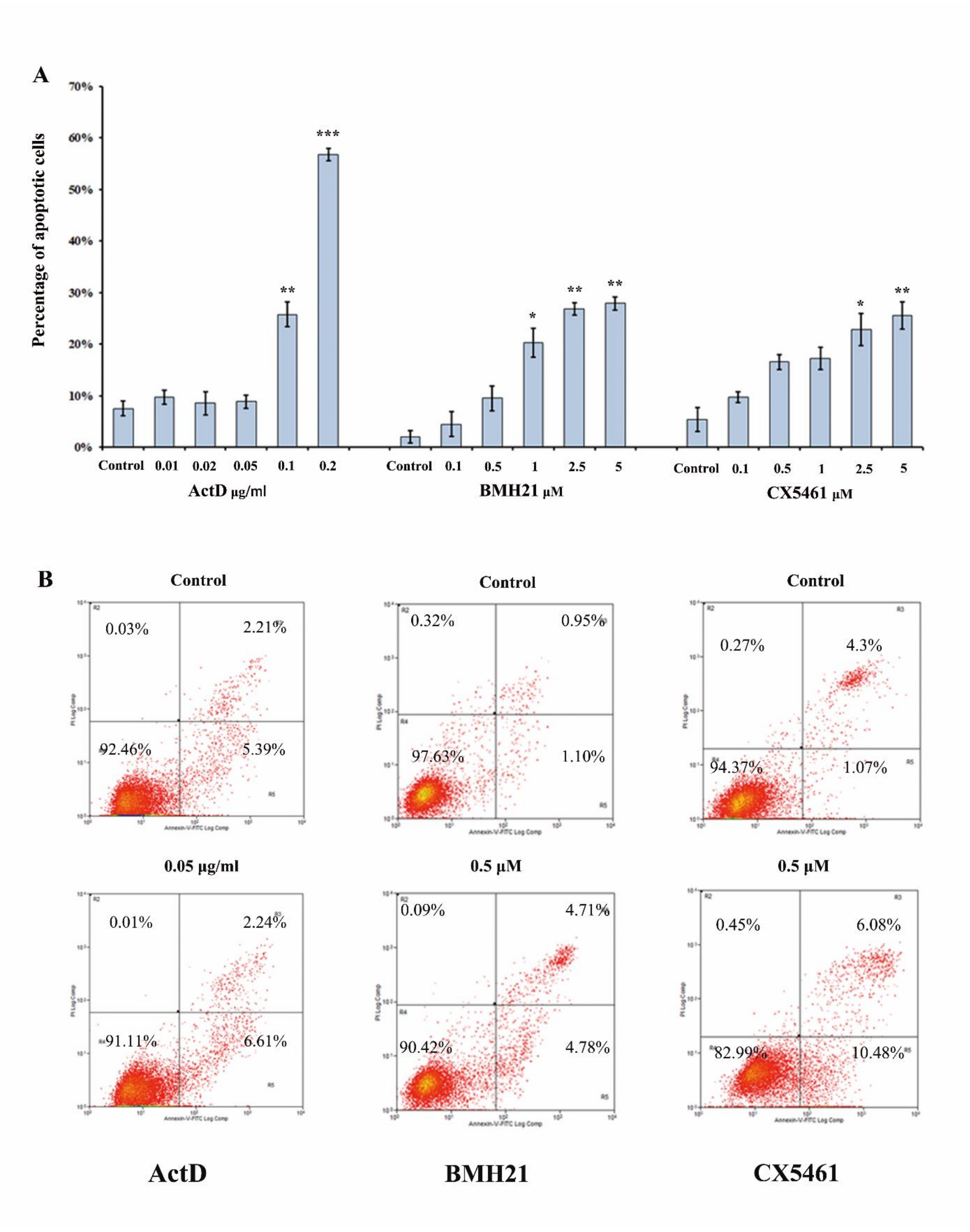

Supplementary Figure 1 Flow cytometry analysis of Annexin-V-FITC and PI stained cells. (A) The percentage of apoptotic HeLa cells after treatment with three rRNA transcription inhibitors ActD, BMH21 and CX5461 at 24 h, respectively. HeLa cells were stained with Annexin-V-FITC and PI and

the numbers of apoptotic cells were counted with flow cytometry. (B) Apoptotic cells were monitored by annexin V-FITC/PI staining and flow-cytometry analysis of HeLa cells treated with 0.05 μg/ml ActD, 0.5 μM BMH21, 0.5 μM CX5461 for 24 h, respectively. The cell stained by Annexin-V-FITC- and PI- was in the lower-left panel; the cell stained by Annexin-V-FITC+ and PI- was in the lower-right panel; the cell stained by Annexin-V-FITC- and PI+ was in the upper-left panel; the cell stained by Annexin-VFITC+ and PI+ was in the upper-right panel. Data are expressed as * $P<0.05$, ** $P<0.01$, *** $P<0.001$, measured by the *t*-test.

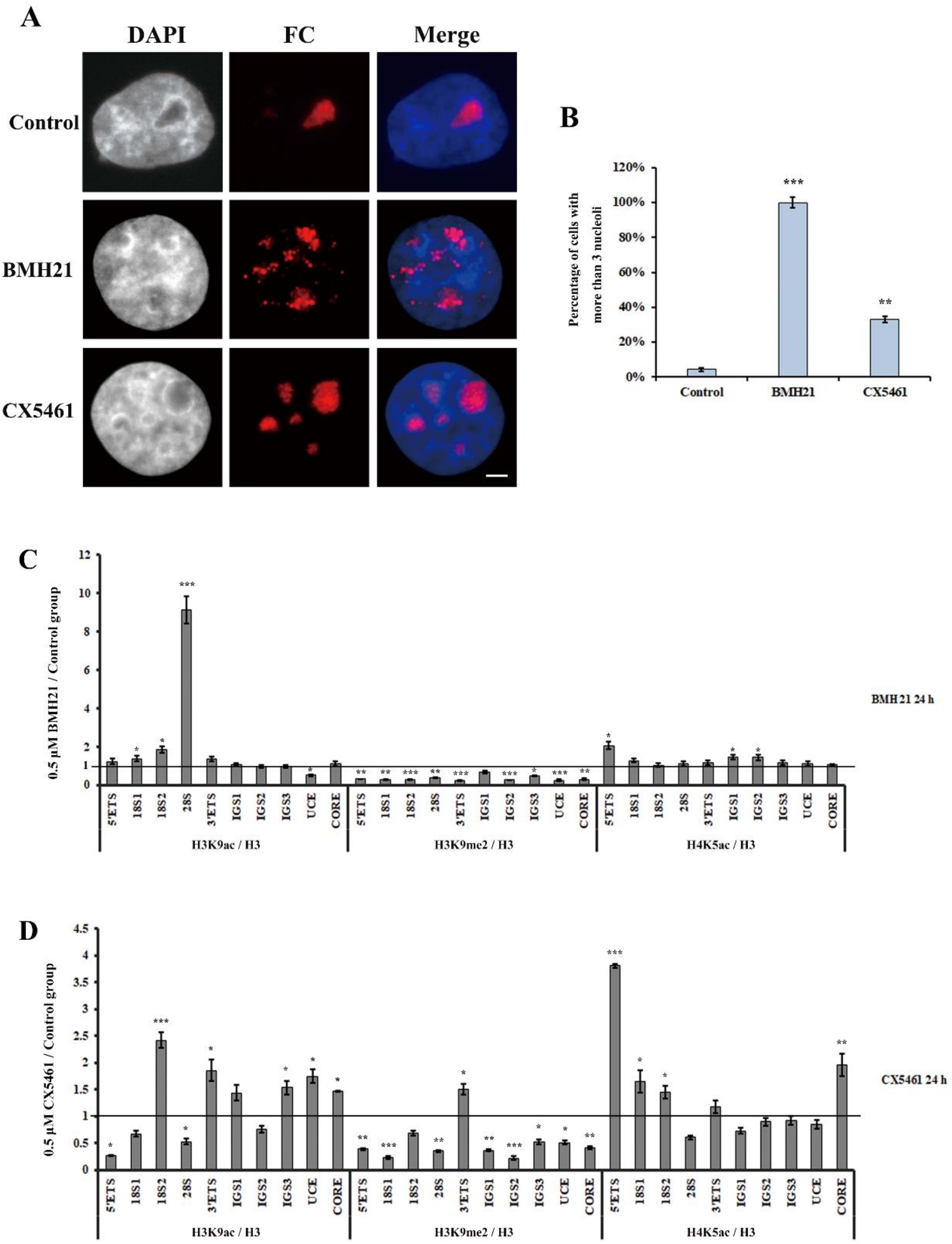

Supplementary Figure 2 effects of rDNA transcription inhibitors in HeLa cells. (A) Nucleoli were detected by indirect immunofluorescence staining with an antibody against nucleolus marker fibrillarin in HeLa cells after treated with BMH21, CX5461 for 24 h, respectively. Bar = 3 μm. (B) Percentages of

interphase nuclei with over three fragmented nucleoli after treatment with or without BMH21, CX5461, respectively. The number of evaluated nuclei in each group was 300. (C-D) ChIP analysis of levels of H3K9ac, H3K9me2, and H4K5ac within rDNA regions in HeLa cells after treatment with or without BMH21, CX5461 for 24 h, respectively. The y-axis indicated the ratio of the relative quantities of DNA in HeLa cells incubated with BMH21, CX5461 to the relative quantities of DNA in untreated HeLa cells. The x-axis indicated different regions of rDNAs. Relative values were normalized to those of the total H3. Each experiment was repeated three times and the average value was shown with the SD. Data are expressed as* P<0.05, ** P<0.01, *** P<0.001, measured by the *t*-test.

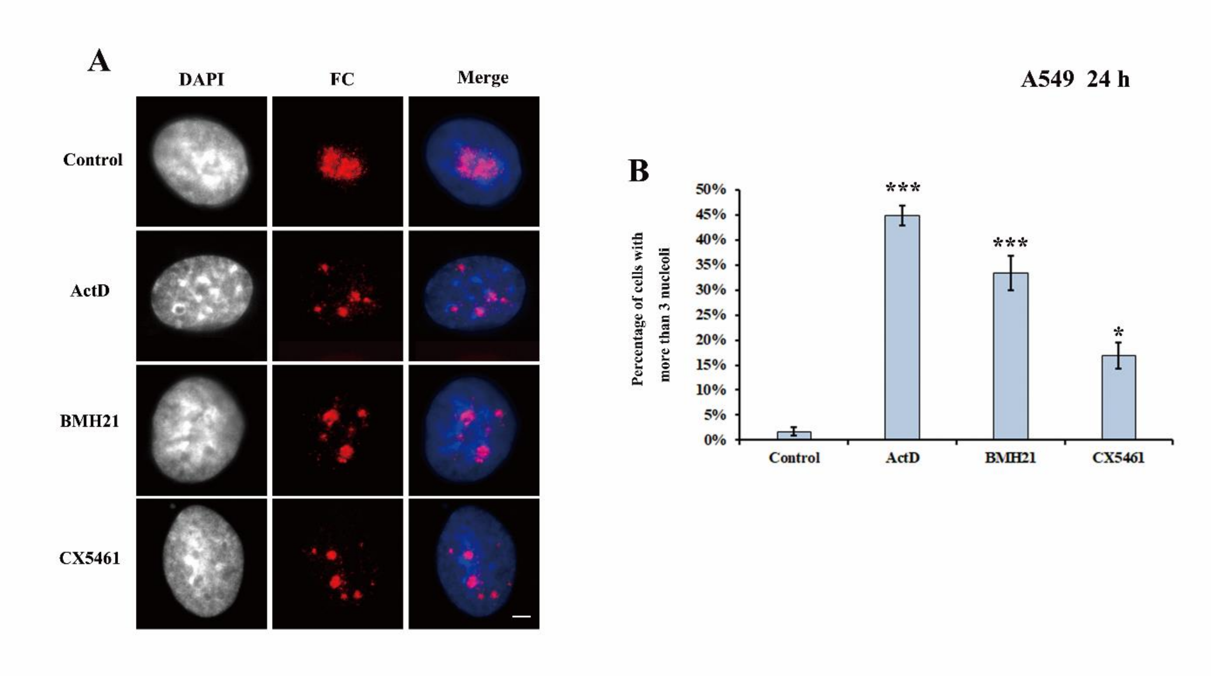

Supplementary Figure 3 Effects of transcription inhibitors in nucleoli of A549 cells. (A) Nucleoli were detected by indirect immunofluorescence staining with an antibody against fibrillarin in A549 cells after treated with ActD, BMH21, CX5461 for 24 h, respectively. Bar = 3 μm. (B) Percentages of interphase nuclei with more than three fragmented nucleoli after treatment with or without ActD, BMH21, CX5461, respectively. The number of evaluated nuclei in each group was 300. Data are

expressed as* $P<0.05$, ** $P<0.01$, *** $P<0.001$, measured by the *t*-test.

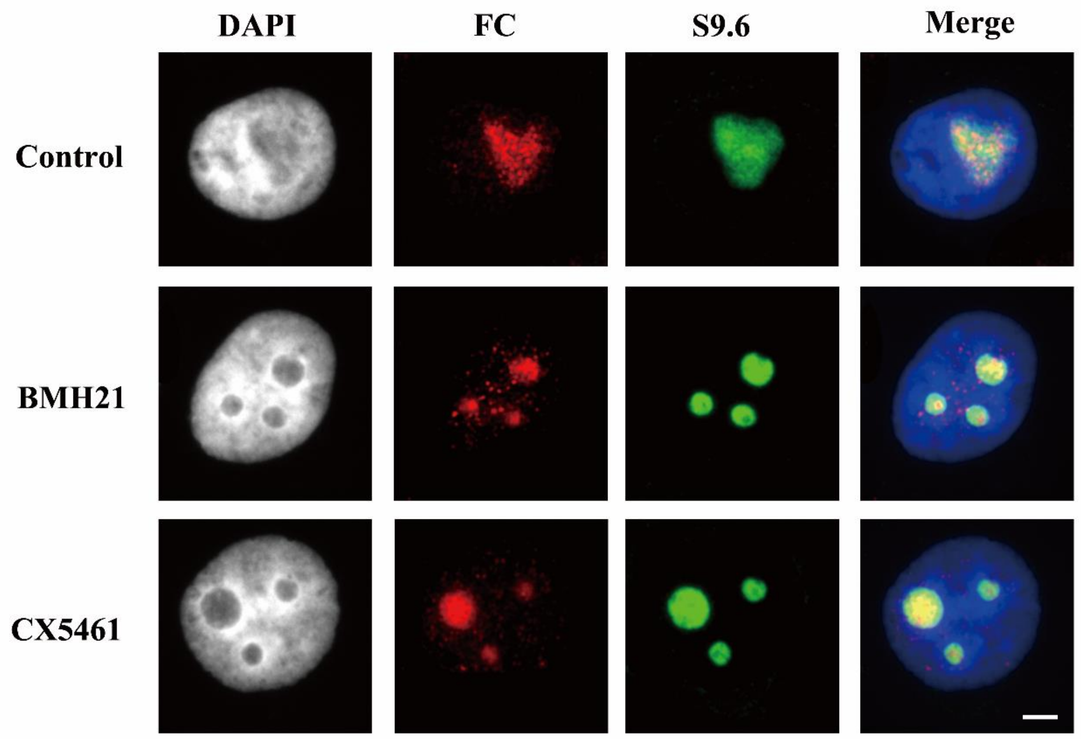

Supplementary Figure 4 Detection of nucleolus and R-loops in interphase nuclei of Hela cells after treatment with BMH21 or CX5461. The nucleolus was stained with the antibody against fibrillarin (FC, red) and R-loops were stained with the antibody against RNA-DNA hybrid (S9.6, green). The upper panel exhibits the interphase nuclei of normal HeLa cells. The middle and lower panels showed interphase nuclei of HeLa cells incubated with BMH21 and CX5461 for 1 h, respectively.

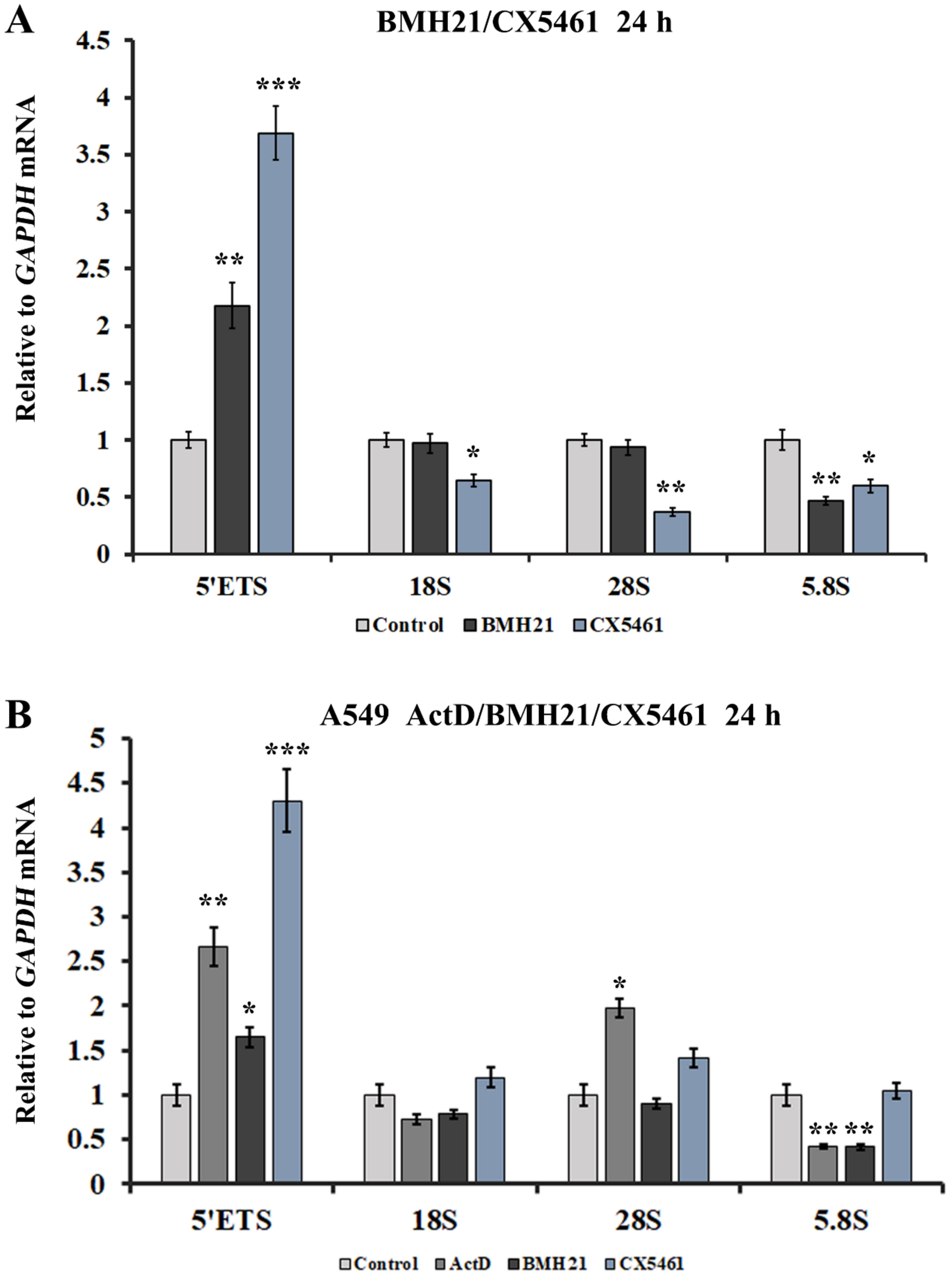

Supplementary Figure 5 Transcription inhibitors effect rDNA transcription initiation in HeLa

and A549 cells. (A) qRT-PCR was used to detect the incomplete 5'ETS transcripts and the mature rRNA expressions in HeLa cells after treated with BMH21 or CX5461 for 24 h, respectively. (B) qRT-PCR was used to detect the incomplete 5'ETS transcripts and the mature rRNA expressions in A549 cells after treated with ActD, BMH21 or CX5461 for 24 h, respectively. Expression values were normalized to the gene *GAPDH*. The relative expression ratio of each sample was compared with untreated cells, expression value of which were assigned as 1. Each experiment was repeated three times, and the average value and SD are shown. Data are expressed as* $P<0.05$, ** $P<0.01$, *** $P<0.001$, measured by the *t*-test.
